# The scaffold protein Tks4 is required for the differentiation of mesenchymal stromal cells (MSCs) into adipogenic and osteogenic lineages

**DOI:** 10.1038/srep34280

**Published:** 2016-10-06

**Authors:** Metta Dülk, Gyöngyi Kudlik, Anna Fekete, Dávid Ernszt, Krisztián Kvell, Judit E. Pongrácz, Balázs L. Merő, Bálint Szeder, László Radnai, Miklós Geiszt, Dalma E. Csécsy, Tamás Kovács, Ferenc Uher, Árpád Lányi, Virag Vas, László Buday

**Affiliations:** 1 Institute of Enzymology, Research Centre for Natural Sciences, Hungarian Academy of Sciences, Budapest, Hungary; 2 Department of Pharmaceutical Biotechnology, Faculty of Pharmacy, University of Pecs, Hungary; 3 Szentagothai Research Center, University of Pecs, Hungary; 4 Department of Physiology, Semmelweis University Medical School, Budapest, Hungary; 5 “Momentum” Peroxidase Enzyme Research Group of the Semmelweis University and the Hungarian Academy of Sciences, Budapest, Hungary; 6 Stem Cell Biology, National Blood Service, Budapest, Hungary; 7 Department of Immunology, Faculty of Medicine, University of Debrecen, Debrecen, Hungary; 8 Department of Medical Chemistry, Semmelweis University Medical School, Budapest, Hungary

## Abstract

The commitment steps of mesenchymal stromal cells (MSCs) to adipogenic and other lineages have been widely studied but not fully understood. Therefore, it is critical to understand which molecules contribute to the conversion of stem cells into differentiated cells. The scaffold protein Tks4 plays a role in podosome formation, EGFR signaling and ROS production. Dysfunction of Tks4 causes a hereditary disease called Frank-ter Haar syndrome with a variety of defects concerning certain mesenchymal tissues (bone, fat and cartilage) throughout embryogenic and postnatal development. In this study, we aimed to analyze how the mutation of Tks4 affects the differentiation potential of multipotent bone marrow MSCs (BM-MSCs). We generated a Tks4 knock-out mouse strain on C57Bl/6 background, and characterized BM-MSCs isolated from wild type and Tks4^−/−^ mice to evaluate their differentiation. Tks4^−/−^ BM-MSCs had reduced ability to differentiate into osteogenic and adipogenic lineages compared to wild type. Studying the expression profile of a panel of lipid-regulated genes during adipogenic induction revealed that the expression of adipogenic transcription factors, genes responsible for lipid droplet formation, sterol and fatty acid metabolism was delayed or reduced in Tks4^−/−^ BM-MSCs. Taken together, these results establish a novel function for Tks4 in the regulation of MSC differentiation.

Frank-ter Haar syndrome (FTHS, OMIM:249420), is a rare genetic disorder associated with skeletal defects, craniofacial anomalies, cardiovascular abnormalities and, in some cases, reduced lipoid tissue^1^,^2^. The majority of FTHS patients die in infancy or in early childhood due to cardiovascular symptoms or respiratory infections^3^. The most common underlying genetic defects in FTHS have been recently identified through homozygosity mapping studies in patients, identifying homozygous mutations in the *SH3PXD2B* gene on chromosome 5q35.1^3^. The analysis of patients detected 4 different intragenic mutations, and one complete deletion of *SH3PXD2*^3^. A novel mutation in FTHS patients has also been described as the deletion of exon 13 of the *SH3PXD2B* gene^4^. Recently, two new homozygous loss-of-function mutations were identified in the *SH3PXD2B* gene in patients with Borrone dermato-cardio-skeletal syndrome (BDSC syndrome) which is a FTHS related genetic disease^5^.

The protein product of the *SH3PXD2B* gene is known as Tks4 (tyrosine kinase substrate with 4 SH3 domains)^6^, a scaffold protein. Upon phosphorylation by Src kinase, it has the ability to interact with signaling molecules to regulate the actin cytoskeleton^7^. Tks4 was also shown to play an important role in the formation of podosomes^8^, production of reactive oxygen species (ROS) by tumor cells^9^, and also involved in EGFR signaling^10^,^11^. Although we have some knowledge of the possible function of Tks4, the detailed mechanism of how Tks4 impacts FTHS affected tissues is less clear.

Mesenchymal stromal cells (MSCs) as multipotent adult stem cells are able to form multiple cell types of mesenchymal origin, e.g. adipocytes and osteoblasts^12^,^13^, therefore it is tempting to speculate that Tks4 may affect osteogenesis and lipogenesis of MSCs. Moreover, there are some hints for the possible role of Tks4 in MSC biology. For example, membrane type-1 matrix metalloproteinase (MT1-MMP), which is a binding partner of Tks4, is known to play a role in MSCs differentiation and trafficking^14^. Moreover, it has been described that Tks4 is involved in ROS production and ROS modulates several signaling pathways regulating MSC differentiation^15^. Therefore, we hypothesized that Tks4 may play a role in the process necessary for MSC differentiation and one of the underlying mechanisms causing the FTHS phenotype could be the impaired stem cell functions of Tks4 deficient MSCs.

Here we present a novel Tks4^−/−^ mouse strain on C57Bl/6 background with the complete loss of Tks4 protein. The adult Tks4 deficient mice have reduced fat tissue mass and altered craniofacial and skeletal bones. We compared the phenotype and differentiation potential of BM-MSCs (bone marrow mesenchymal stromal cells) isolated from Tks4^−/−^ and wild type mice. Our data demonstrate that in the absence of Tks4, adipogenic and osteogenic differentiation of BM-MSCs is impaired; therefore, we concluded that Tks4 is necessary for the adipogenic and osteogenic mesenchymal differentiation pathways.

## Results and Discussion

### Description of a novel Tks4 null mouse

**Figure 1:**
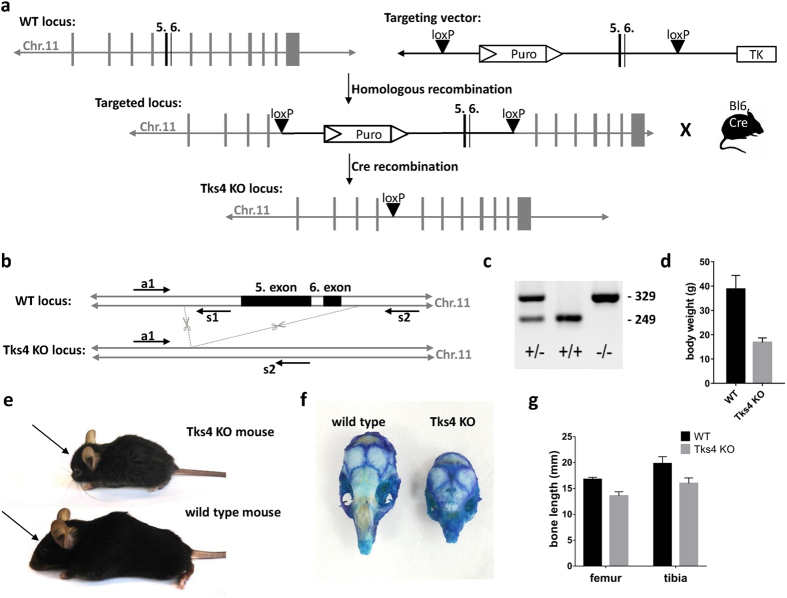
Generation and characterization of Tks4-deficient mice. (**a**) Gene targeting strategy to knock out exons 5 and 6 of Tks4. In the targeting vector, exons 5 and 6 were flanked by loxP sites. A puromycin (Puro) resistance gene cassette was inserted into intron 4 and the thymidine kinase gene (TK) was inserted downstream of exon 6 for positive and negative selection, respectively. Mice carrying the mutant floxed allele were crossed with transgenic C57Bl/6 mouse carrying Cre recombinase. (**b**) Position of deleted exons 5 and 6 are depicted in chromosome 11. The primer set (a1, s1, s2) and the amplified regions (WT: 249 bp, KO: 329 bp) are indicated on the *SH3PXD2B* wild type (WT) and knock-out (KO) gene. (**c**) PCR genotyping of heterozygous (^+/−^), wild type (^+/+^) and homozygous Tks4 knock-out (^−/−^) mice. Genomic DNAs obtained from offspring of heterozygous (^+/−^) mice, were amplified using primer sequences (a1, s1, s2) located near the deleted region. (**d**) Body weights of 8–10 months old Tks4^−/−^ mice (n = 4) and wild type mice (n = 3). (**e**) Tks4^−/−^ mouse and wild type littermate. Arrows show the shorter nasal bone of Tks4^−/−^ mouse compared to wild type. (**f**) Calvarias from an 8 months old wild type and a littermate Tks4^−/−^ mouse were stained with methylene blue. (**g**) Bone length measurements of 8–12 months old Tks4^−/−^ mice (n = 5) and wild type mice (n = 5). *p < 0.05. An unpaired t-test was used to determine the significance of the difference between means of two groups. Error bars represent s.d.

**Figure 2:**
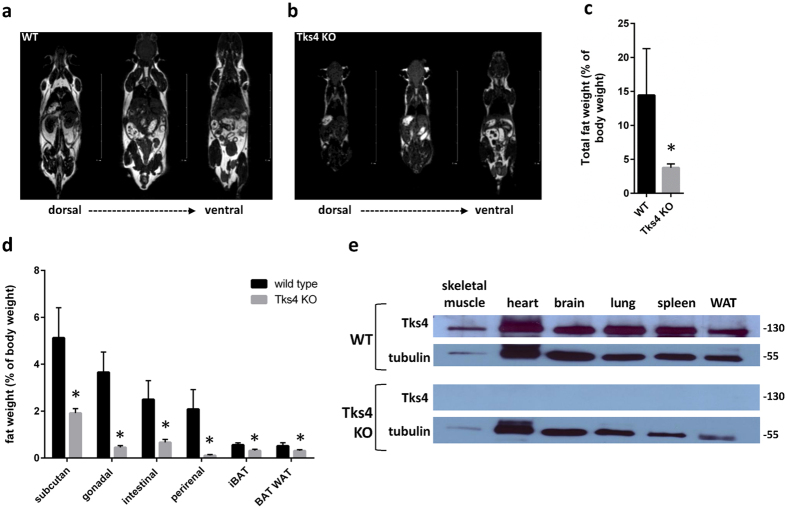
Characterization of Tks4-deficient MSCs. MRI measurement showing fat tissues (white) and other tissues (gray or black), (**a**) represents a 7 months old wild type male mouse and (**b**) represents a 7 months old Tks4 deficient male mouse. (**c**) Total fat weight measured in three adult WT and Tks4^−/−^ mice. (**d**) Weights of various fat depos isolated from 7 months old WT and Tks4 KO mice. Three adult mice in each group were analyzed. (**e**) Skeletal muscle, brain, heart, lung, WAT (gonadal white adipose tissue) and spleen lysates from WT and KO mice were analyzed by Western blot for Tks4. Samples and gels were handled and run under the same experimental conditions. Tubulin was used to control equal loading. *p < 0.05. An unpaired t-test was used to determine the significance of the difference between means of two groups. Error bars represent s.d.

Using homologous recombination with the targeting vector described in Fig. 1a, we have generated mutant mice in which the fifth and sixth coding exons of the *SH3PXD2B* gene were flanked by loxP sites and the puromycin resistance gene cassette was inserted into intron 4 adjacent to the floxed exons (Fig. 1a–c). Although Tks4^−/−^ mice are viable and yielded the expected female-to-male ratio, they are infertile. The major observed phenotypic consequences of the genetic deletion of Tks4 scaffold protein were smaller size compared to the wild type (Fig. 1d), craniofacial abnormalities with wide anterior fontanel and prominent eyes, (Fig. 1e,f) skeletal malformations as shortened long bones and kyphosis (Fig. 1g and Supplementary Table S1). MRI measurements showed that the adult Tks4 null mice develop lipodystrophy (Fig. 2a,b). Dissection of the mice showed highly reduced total fat weight and the separate fat pads of subcutaneous, gonadal, intestinal, and perirenal white adipose tissue also decreased in size in Tks4^−/−^ mice compared to wild types (Fig. 2c,d). The phenotype of our Tks4^−/−^ C57Bl/6 mice is similar to other Tks4^−/−^ mice established in other mouse strains^3^,^16^ (Supplementary Table S1) and resembles the symptoms of FTHS patients^2^. To study the expression of Tks4 protein in wild type C57Bl/6 mice and confirm the lack of Tks4 protein in KO mice, we selected skeletal muscle, brain, heart, lung, spleen and white adipose tissue for Western blot examination (Fig. 2e). Tks4 protein was present in all wild type mouse tissue samples. High level of Tks4 expression was also measured in white adipose tissue. However, as expected, Tks4 was completely missing in the tissues isolated from KO mice.

### Characterization of mesenchymal stromal cells of the Tks4-null mice

The major disease-affected tissues in FTHS patients and in Tks4^−/−^ mice are bones, cartilage and fat tissue, all with mesenchymal origin. MSCs have the ability to differentiate into cells of mesenchymal lineage, but the involvement of Tks4 protein in the MSC differentiation process has not been tested so far.

**Figure 3:**
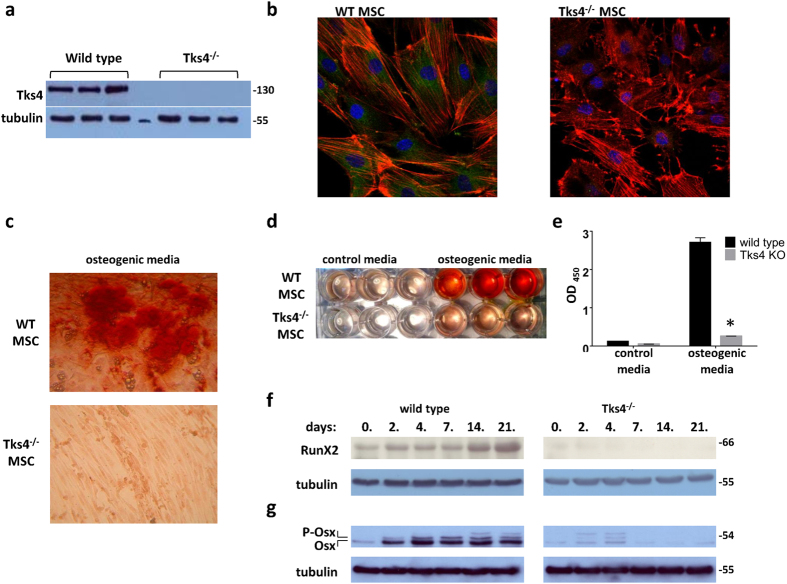
Altered osteogenic differentiation potential of Tks4^−/−^ BM-MSCs. (**a**) Western blot analysis of cell extracts from three independent wild type and three independent Tks4 KO BM-MSC lines. Protein samples were subjected to SDS-PAGE followed by immunoblots with anti-Tks4. Tubulin was used to control equal loading. (**b**) Representative fluorescent images of wild type and Tks4 knock-out cell cultures. BM-MSCs were grown in confocal chambers, fixed, permeabilized and stained. Nuclei were stained with DAPI (blue), actin filaments were fixed/labeled with Phalloidin-TRITC (red) and anti-Tks4 was visualized with Alexa Fluor 488 (green). (**c**) Wild type and Tks4^−/−^ MSCs were incubated in ODM and pictures were taken after Alizarin Red S staining. Representative pictures of stained cultures in 10x magnification following 14 days of culture, showing the morphology of the cells and (**d**) the whole plate showing the differences between control and ODM treatment of the Tks4^−/−^ and wild type MSC cultures. (**e**) Quantification of calcium deposition by detecting the absorbance of Alizarin Red S extracts. Data are represented as mean of OD values of three wells. Time course of (**f**) RunX2 and (**g**) Osterix (Osx) osteogenic marker expressions during *in vitro* osteogenic differentiation of wild type and Tks4^−/−^ BM-MSCs. (**g**) The phosphorylated form of Osterix (P-Osx) appears as lower mobility bands. Cell lysates were prepared at various time points and Western blot analyses were performed. Gels were run simultaneously under the same experimental conditions. Tubulin was used to control equal loading. *p < 0.05. An unpaired t-test was used to determine the significance of the difference between means of two groups. Error bars represent s.d.

Therefore, we studied the expression of Tks4 in BM-MSCs and the necessity of its presence in the BM-MSC differentiation process. First, we isolated MSCs from bone marrow of wild type and Tks4 mutant mice as described in previous reports^17^,^18^. The phenotype of fibroblastic, plastic adherent BM-MSCs was evaluated to confirm the identity of the cells by flow cytometry. Immunophenotyping showed that the Tks4^−/−^ and wild type BM-MSC were negative for certain cell-surface antigens common to myeloid and lymphoid lineages, including CD45, F4/80; but were positive for markers used to describe mouse MSCs such as Sca-1 and CD44 and express CD73 and CD90. The cell surface antigen expression was similar in case of wild type and the Tks4^−/−^ BM-MSCs and comparable to other mouse mesenchymal stromal cell lines^19^,^20^ (Supplementary Fig. S1). MSCs have not been tested so far for the presence of Tks4 protein; therefore, we checked the expression of Tks4 in all isolated BM-MSC lines. As shown in Fig. 3a, all three wild type MSCs expressed Tks4, whereas it was missing from Tks4^−/−^ cells. To visualize Tks4 in BM-MSCs, wild type and Tks4^−/−^ cells, we used immunofluorescence staining and microscopy. Figure 3b demonstrates that Tks4 displays a cytoplasmic expression pattern in wild type BM-MSCs, while it is missing from Tks4^−/−^ cells. It is worth noting that the majority of wild type MSCs displays actin stress fibers while they are not present in a noticeable portion of Tks4^−/−^ cells where F-actin appears rather around the cell periphery. This observation is in line with the fact that Tks4 is able to bind and recruit actin regulators; therefore, actin filaments are differently distributed in the presence than in the absence of Tks4^6^.

### Tks4 is a potential regulator of the adipogenic and osteogenic differentiation of BM- MSCs

To test the BM-MSC multipotent capacity, these cells were first subjected to osteogenic differentiation condition. As seen in the Alizarin Red S-stained cultures (Fig. 3c), Tks4 mutant MSCs were not able to deposit calcium-containing minerals and remained elongated cells. In contrast, wild type MSCs readily differentiated into hydroxyapatite-depositing osteoblast cells. The photometric quantification of Alizarin Red S stain accumulation in cultures of differentiated MSCs also confirmed the reduced ability of Tks4^−/−^ MSCs to differentiate into osteogenic lineage (Fig. 3d,e). To characterize the differentiation defect of Tks4 KO BM-MSCs, we examined the expression of the main osteogenic transcription factors, RunX2 (66 kDa) and Osterix (54 kDa) by western blot (Fig. 3f,g)^21^. RunX2 in wild type BM-MSCs was considerably upregulated during osteogenic differentiation, as expected^22^,^23^. By contrast, the expression of RunX2 in Tks4^−/−^ BM-MSCs was lower and decreased throughout the differentiation process (Fig. 3f). As shown on Fig. 3g, Osterix was present during the differentiation of wild type MSCs, moreover the expressed Osterix protein became noticeably phosphorylated^24^. Meanwhile, the Tks4 mutant MSCs displayed reduced Osterix expression and weak phosphorylation, indicating that the transactivator activity of the protein is not upregulated^24^.

The facts, that RunX2 and Osterix have decreased expression levels in the Tks4^−/−^ MSCs compared to wild type support the conclusion that osteogenic differentiation of Tks4 mutant MSCs is impaired.

**Figure 4:**
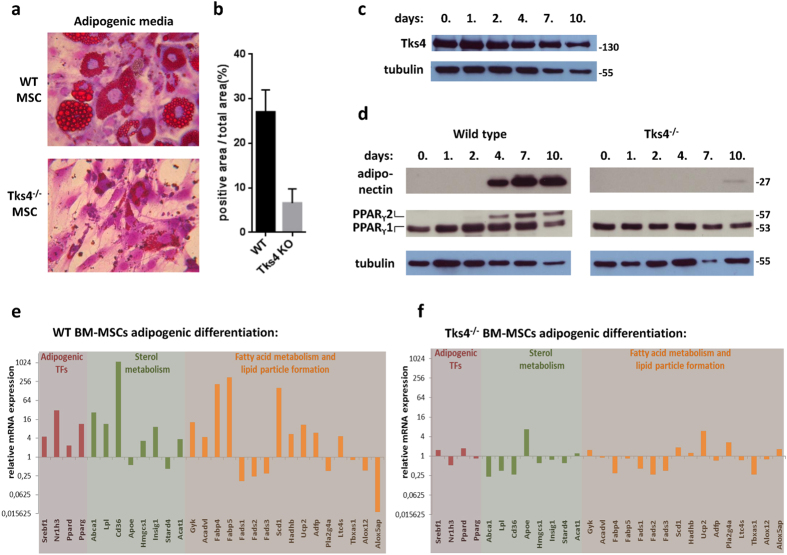
Reduced adipogenic differentiation potential of Tks4^−/−^ BM-MSCs. *In vitro* adipogenesis of MSCs isolated from bone marrow of wild type and Tks4^−/−^ mice. (**a**) Representative Oil red O-stained cultures and (**b**) the quantification of Oil Red O content (n = 8) following differentiation for 7 days. (**c**) Time course of Tks4 protein expression in wild type MSCs during adipogenic differentiation. Cell lysates were prepared at various time points and Western blot analyses were performed with anti-Tks4 antibody. Tubulin was used to control equal loading. (**d**) Time course of adiponectin and PPAR_ϒ_ expression during *in vitro* adipocyte differentiation of wild type and Tks4^−/−^ BM-MSCs. Cell lysates were prepared at various time points and Western blot analysis were performed. Tubulin was used to control equal loading. Gels were run simultaneously under the same experimental conditions. The adipo-differentiated wild type (**e**) and Tks4^−/−^ MSCs (**f**) were subjected to a TaqMan array for mouse lipid-regulated genes and gene expression profile was analyzed. The mRNA levels measured for day 0 of differentiation (control) were set to 1. The mRNA levels measured for day 4 of differentiation are calculated as n-fold differences relative to the control (day 0) samples. The relative expression levels of each gene are shown. (**e**,**f**) Indicates genes at least 2-fold up- or downregulation^29^.

Next, we tested the adipogenic potential of Tks4^−/−^ MSCs. BM-MSCs were kept in adipogenic medium (ADM) and the differentiated cells were detected by Oil red O staining. After 7 days of culture, the wild type MSCs developed lipid droplets in the cytoplasm but the Tks4 deficient MSCs did not show substantial lipid accumulation (Fig. 4a,b). These results revealed that the Tks4^−/−^ MSCs were unable to undergo the adipogenic differentiation as efficiently as the wild type MSCs.

In the following experiments, we focused on adipogenic differentiation and further studied the functional relationship between Tks4 and MSC differentiation. As a starting point, we confirmed the expression of wild type Tks4 protein during adipogenic differentiation. Along the course of adipogenic induction of wild type MSCs, cell lysates were collected from cultures at six time points until day 10 and immunoblots were performed for Tks4 (Fig. 4c). Our results showed that Tks4 protein is continuously present at all time points measured. This is in line with a previous study where the adipogenic differentiation of 3T3-L1 cell line was tested and the mRNA of Tks4 was present in the early stage of adipocyte differentiation^25^.

Expression of a number of regulatory and structural proteins is required for the proper adipose tissue differentiation, including PPARγ and adiponectin^26^,^27^. PPARγ has two isoforms, PPARγ1 and PPARγ2, generated by alternative promoter usage and splicing. Compared with the isoform 1, PPARγ2 contains an N-terminal 30-amino-acid extension and displays lower mobility on SDS-PAGE^28^. We investigated the expression of PPARγ and adiponectin during the differentiation process of MSCs. Immunoblot analysis with anti-PPARγ antibody revealed constitutive PPARγ1 expression in wild type MSCs, while PPARγ2 appeared around day 4 in the cells (Fig. 4d). Tks4^−/−^ MSCs also contained PPARγ1 at all time points but PPARγ2 expression could not be detected. Figure 4d also demonstrates that adiponectin was readily detectable around day 4 in adipocytes derived from wild type MSCs, while Tks4 deficient cells only started to express adiponectin at low level around day 10. These results suggest that the presence of Tks4 is necessary for adipocyte differentiation and in the absence of Tks4, the adipogenic differentiation is blocked or delayed.

To further confirm the involvement of Tks4 protein in the adipogenic differentiation, the gene expression profile of a panel of lipid-regulated genes was measured. Wild type and Tks4^−/−^ MSCs were incubated in adipogenic media for 4 days and subjected to a Taqman Array for mouse lipid-regulated genes^29^,^30^ (Supplementary table S2). The analysis revealed that the mRNA levels of transcription factors related to adipocyte differentiation (e.g. Srebf1, Nr1h3, PPARγ) increased in wild type adipogenic MSC cultures but did not change significantly in Tks4 KO samples (Fig. 4e,f). Furthermore, the mRNA levels of most enzymes involved in sterol-metabolism, including Abca1, Lpl, Cd36 were down-regulated in the Tks4^−/−^ MSCs in ADM. Moreover, genes characteristics for fatty acid synthesis, such as Fabp4, Fabp5, Scd1 significantly increased in wild type samples but remained almost unchanged in Tks4^−/−^ adipogenic MSC cultures. Taken together, we could measure significant up-regulation of 19 lipid-regulated genes in wild type adipogenic MSC cultures but the expression of these genes did not change dramatically in the Tks4^−/−^ MSC samples. These results show that ADM has an inductive effect on several genes important for lipid metabolism in wild type MSCs but the lipid-regulated gene transcriptome remains rather unaffected in Tks4^−/−^ MSCs during the culturing period.

## Conclusions

The results presented here demonstrate that the scaffold protein Tks4 plays an important role in the differentiation process of BM-MSCs into adipogenic and osteogenic lineages. Our finding that the differentiation of MSCs is impaired in the absence of Tks4 may serve an explanation for why Tks4 deficient mice and Frank-ter Haar syndrome patients display such a compromised phenotype. Future studies are warranted to describe the precise mechanism of Tks4 scaffold protein in the signaling of stem cell differentiation.

## Material and Methods

### Generation of Tks4 knock-out mice

Tks4 knock-out mice were generated by targeted disruption of *SH3PXD2B* gene on chromosome 11, followed by the germline transmission of the mutated gene in Tks4^+/−^ and Tks4^−/−^ mice by TaconicArtemis. In the targeting vector (designed to allow a conditional knock-out of Tks4), the fifth and sixth coding exons of the *SH3PXD2B* gene were flanked by loxP sites and a puromycin resistance gene cassette was inserted into intron 4 adjacent to the floxed exons. (Figure 1a) The thymidine kinase gene was placed next to the homologous sequence for negative selection. The C57BL/6N ES cell line was grown on a mitotically inactivated feeder layer comprised of mouse fibroblasts in DMEM high glucose medium containing 20% FBS and 1200 U/ml Leukemia Inhibitory Factor. 1 × 10^7^ cells and 30 μg of linearized DNA vector were electroporated (Biorad Gene Pulser) at 240 V and 500 μF. Puromycin selection (1 μg/ml) started on day 2, counter selection with Gancyclovir (2 μM) started on day 5 after electroporation. ES clones were isolated on day 8 and analyzed by Southern blotting according to standard procedures after expansion and freezing of clones in liquid nitrogen.

The floxed fifth and sixth exons were removed by cre-mediated recombination in the germ line. Genotyping was performed by PCR using oligonucleotide primers a1 vs. s1 (a1: ACC CCA TAT CCA AAT TGT TGG and s1: GGA TAC ATT ATG CTG GCA TCG) for the wild type allele (product size, 249bp) and oligonucleotides a1 vs. s2 (s2: GGT TTG AGT GAC AGG TAT CAA CC) for the mutant reaction (product size, 329 bp). (Figure 1b) Inactivation of the SH3PXD2B gene was tested in every generation by PCR of genomic DNA. (Figure 1c) Animal procedures in this study were conducted under the approval of the Institutional Animal Ethics Committee. (Approval number: 22.1/2236/003/2009). The animals were maintained and handled in accordance with the Guidelines for Accommodation and Care of Animals (European Convention for the Protection of Vertebrate Animals Used for Experimental and Other Scientific Purposes).

### Generation of BM-MSC cultures

We used 10–12 weeks old C57Bl/6 wild type and C57Bl/6 Tks4^−/−^ mice for the generation of wilde type and KO BM-MSC cultures. Methods were performed as described previously^17^,^18^. Briefly, the mice were euthanized and femurs and tibiae were collected in Petri dishes containing Hank’s balanced salt solution (HBSS) (Invitrogen, Carlsbad, CA, USA). After clearing the bones from excess tissues and removing the epiphyses, bone marrow nucleated cells were flushed out with complete medium (CM) containing DMEM/Ham’s F-12 medium (Thermo Fisher Scientific, Bremen, Germany), 10% fetal bovine serum, 5% horse serum (Invitrogen), 50 U/ml penicillin, 50 μg/ml streptomycin (Sigma-Aldrich, St Louis, MO, USA) and 2 mM L-glutamine (Invitrogen) supplemented with heparin at a final concentration of 5 U/ml. The suspension was washed twice with HBSS then cells were seeded into 25 cm^2^ culture flasks (BD Falcon, Bedford, MA) in CM with a cell density of 1–2 × 10^5^ cm^2^ and placed in a humidified incubator at 5% CO_2_ and 37 °C. After two days, media were changed (fresh CM) and thus non-adherent cells were removed. Cells were propagated by trypsinization (0.25% Trypsin-EDTA solution (Invitrogen)) upon reaching confluence and were placed into 75 cm^2^ culture flasks (BD Falcon). From the second passage, cells were subcultured in a 1:5 ratio and were used from passage 4 to 11 in our experimental settings. We have repeated the MSC isolation three times to ensure true biological parallels of the further analysis. In each time points, the bone marrow of three wild type mice and three Tks4^−/−^ mice were pooled and used to establish WT MSC and Tks4 KO MSC lines.

### Characterization of BM-MSCs by flow cytometry

To define WT and Tks4 KO BM-MSCs by cell surface antigens, cells were retrieved by trypsinization (0.25% Trypsin-EDTA) at passages 3., 6. and 9 for flow cytometry analysis. Suspensions containing 1 × 10^5^ cells were made and labelled for 20 minutes in the dark at 4 °C with either fluorescein isothiocyanate- or phycoerythrin-conjugated monoclonal antibodies against mouse Sca-1, CD44, CD73, CD90.2 (all from BD Pharmingen, San Diego, CA, USA), CD45R, and F4/80 (AbD Serotec Ltd., Oxford, UK). Fluorescence was detected by flow cytometry using an Attune flow cytometer (Life Technologies, Carlsbad, CA) and the corresponding Attune Cytometer Software. Data are represented with the appropriate isotype controls.

### Adipogenic and osteogenic differentiation of BM-MSCs

Wild type and Tks4 KO BM-MSCs were seeded into 24-well flat bottom plates or 25 cm^2^ culture flasks (all BD Falcon) and grown in CM. CM was replaced by the appropriate differentiation media upon reaching confluence or cells were harvested on day 0 as control sample for further analysis. Adipogenic differentiation medium (ADM) contained DMEM/Ham’s F12 supplemented with 10% FCS, 0.5 mM 3-isobutyl-1-methylxanthine (IBMX; Sigma-Aldrich) and 0.1 μM dexamethasone (Sigma-Aldrich) while osteogenic differentiation medium (ODM) consisted of DMEM (Thermo Fisher Scientific) supplemented with 10% FCS, 10 mM β-glycerophosphate (Sigma-Aldrich), 50 μg/ml ascorbic acid (Sigma) and 0.01 μM hydrocortisone (Sigma-Aldrich). During differentiation, ADM or ODM were replaced every 3–4 days. Cultures were kept in ADM for 1–10 days and the accumulation of lipid droplets was assessed by fixing cells with 8% formalin for 20 minutes at 4 °C and staining with Oil Red O (Sigma Aldrich) and dimethylmethylene blue. Osteogenic differentiation was proved after 14 days in ODM. Cells were fixed with 8% formalin and extracellular calcium deposition was stained with Alizarin Red S (Sigma Aldrich). In order to quantify adipogenic differentiation potential, photographs were taken of the Oil Red O stained cultures with a digital camera (Nikon Coolpix 4500, Tokyo, Japan) connected to an inverted microscope (Olympus CK2) and stain accumulation was quantified according to Deutsch *et al.*^31^. To quantify osteogenic differentiation potential, Alizarin Red S-stained dry 24-well flat bottom plates were treated with 0.5 ml/well extraction solution of 20% (v/v) methanol and 10% (v/v) acetic acid in distilled water. Photographs were taken at this step of the whole plate, and then 100–100 μl of the resulted solutions from each parallel wells were transferred to a 96-well plate (BD Falcon) and the optical densities (OD) at 450 nm were measured with a photometer.

### RNA isolation, cDNA synthesis

Cultured and differentiated wild type and Tks4 KO BM MSC cells were harvested and pelleted followed by total RNA isolation using the Nucleospin RNA kit according to the manufacturer’s instructions (ref. no.: 740955, Macherey-Nagel). In-kit on-column DNase-treatment was included to eliminate potential genomic DNA contamination. 150 ng total RNA was used for reverse transcription during cDNA synthesis employing the Superscript kit following the manufacturer’s instructions (part no.: 4368814, Life Technologies). The kit includes random hexamer primers and performs first strand synthesis without additional RNase inhibition.

### TaqMan Lipid-Regulated gene expression analysis

96-well plates for Taqman Array of mouse lipid-regulated genes were used for precise gene expression profiling following the manufacturer’s instructions (part no.: 4415461, Life Technologies). The plate allowed for the direct comparison of control and treated samples on the same plate with 2 × 48 well layouts, and also for the parallel use of multiple housekeeping genes (actin, GAPDH, HPRT1, GUSB) for precise standardization at all abundance levels. Plate PCR was run on an HT7500 platform and evaluated using SDS 7500 v2.3 software (Life Technologies). The measured fold increase or decrease of the differentiated BM-MSC samples was normalized to the day 0 control samples. Data were represented in logarithmic scale. The gene set included in the array was grouped according to gene ontology and clustered as transcription factors, sterol metabolism, fatty acid metabolism and lipid droplet formation groups^30^.

### Western blot analysis

Cells were washed with PBS and lysed in ice-cold 30 mM Tris buffer (pH 7.5), containing 100 mM NaCl, 1% Triton X-100, 10 mM NaF, 1 mM EGTA, 1 mM Na3VO4, 2 mM p-nitrophenyl-phosphate, 10 mM benzamidine, 1 mM phenylmethylsulphonyl fluoride (PMSF), 25 μg/ml each of Pepstatin A, trypsin inhibitor and aprotinin. Lysates were clarified by centrifugation at 14000 rpm for 10 min at 4°C. Sample buffer (4x, 0.2 M Tris, 0.277 M SDS, 40% (V/V) glycerol, 0.588 M β-mercaptoethanol, 0.05 M EDTA, 1.19 mM bromophenol blue in distilled water) was then added to the supernatants, and the samples were boiled for 3 min. Equal amounts of samples were subjected to SDS-PAGE using 7.5, 10 or 12.5% running gels, respectively. In case of testing for the presence of Tks4, proteins were transferred to nitrocellulose membranes while in the other cases, PDVF membranes were used. Membranes were blocked and incubated for 60 min with the appropriate primary antibodies at room temperature. Polyclonal anti-SH3PXD2B specific antibody was generated earlier^7^. Anti-α-tubulin (DM1A) was obtained from Sigma-Aldrich, St. Louis, MO, USA, PPARγ (81B8), adiponectin (C45B10) from Cell Signaling Technology, Danvers, MA, USA and anti-RunX2 (ab76956), anti-Osterix (ab22552) from Abcam, Cambridge, UK. After several washing steps, membranes were incubated for 30 min with a horseradish peroxidase-conjugated secondary antibody (GE Healthcare, Little Chalfont, Buckinghamshire, UK) and washed again. Reacting antigens were visualized with the enhanced chemiluminescence (ECL) detection reagents (Amersham Life Sciences Limited, Buckinghamshire, UK).

### Confocal microscopy

Cells plated on μ-Slide 8-well ibiTreat Microscopy Chamber (ibidi GmbH, Martinsried, Germany) were fixed in 4% paraformaldehyde in PBS for 10 min, washed with 0.1% Triton X-100 in PBS and blocked with 2.5% FBS in PBS for 30 min. Anti-Tks4 polyclonal rabbit antibody^7^ was applied at 1:1000 and TRITC-Phalloidin (P1951, Sigma-Aldrich, St. Louis, MO, USA) was applied at 1:500 dilution for 1 h. After washing with 0.05% Triton X-100 in PBS, the sample was incubated with Alexa Fluor 488 labeled anti-rabbit secondary antibody (Molecular Probes®, Thermo Fisher Scientific, Waltham, MA USA) for 30 min at 1:1000 dilution. The pictures of fixed samples were acquired on a Zeiss LSM710 inverted confocal microscope with 63× objective (Carl Zeiss, Jena, Germany). Images were processed using ZEN software (Carl Zeiss, Jena, Germany).

### Positron emission tomography/magnetic resonance imaging (PET/MRI)

Information on adipose tissues obtained by MRI measurement was collected using NanoScan PET/MRI system (Mediso Ltd, Hungary). Mice were anesthetized with isoflurane and underwent MRI without using contrast material. Parameters used: scan range 100 mm, 250 slice, slice thickness 0,4, FO,V 50, matrix 128 × 128, NEX 3, TR/TE/FA 4.4/1.5/60 FESS were allowed.

## Statistical Analysis

All data were analyzed using Prism software and represented as mean and standard deviation (s.d.). Statistical significance was assessed by unpaired Student’s t-tests. Three different wild type and three different Tks4^−/−^ MSC cell lines were established and subjected to all experiments and analysis.

## Additional Information

**How to cite this article**: Dülk, M. *et al.* The scaffold protein Tks4 is required for the differentiation of mesenchymal stromal cells (MSCs) into adipogenic and osteogenic lineages. *Sci. Rep.* **6**, 34280; doi: 10.1038/srep34280 (2016).

## Supplementary Material

Supplementary Table S1

Supplementary Table S2

Supplementary Figure S1

## References

[b1] Frank Y. *et al.* Megalocornea associated with multiple skeletal anomalies: a new genetic syndrome? J. Génétique Hum. 21, 67–72 (1973). 4805907

[b2] Maas S. M., Kayserili H., Lam J., Apak M. Y. & Hennekam R. C. M. Further delineation of Frank-ter Haar syndrome. Am. J. Med. Genet. A. 131, 127–133 (2004). 15523657 10.1002/ajmg.a.30244

[b3] Iqbal Z. *et al.* Disruption of the podosome adaptor protein TKS4 (SH3PXD2B) causes the skeletal dysplasia, eye, and cardiac abnormalities of Frank-Ter Haar Syndrome. Am. J. Hum. Genet. 86, 254–261 (2010). 20137777 10.1016/j.ajhg.2010.01.009 PMC2820172

[b4] Bendon C. L. *et al.* Frank-ter Haar syndrome associated with sagittal craniosynostosis and raised intracranial pressure. BMC Med. Genet. 13, 104 (2012). 23140272 10.1186/1471-2350-13-104 PMC3532175

[b5] Wilson G. R. *et al.* Mutations in SH3PXD2B cause Borrone dermato-cardio-skeletal syndrome. Eur. J. Hum. Genet. doi: 10.1038/ejhg.2013.229. (2013). PMC4023207 24105366

[b6] Buschman M. D. *et al.* The Novel Adaptor Protein Tks4 (SH3PXD2B) Is Required for Functional Podosome Formation. Mol. Biol. Cell 20, 1302–1311 (2009). 19144821 10.1091/mbc.E08-09-0949 PMC2649273

[b7] Lányi Á. *et al.* The homolog of the five SH3-domain protein (HOFI/SH3PXD2B) regulates lamellipodia formation and cell spreading. PloS One 6, e23653 (2011). 21886807 10.1371/journal.pone.0023653 PMC3160312

[b8] Courtneidge S. A. Cell migration and invasion in human disease: the Tks adaptor proteins. Biochem. Soc. Trans. 40, 129–132 (2012). 22260678 10.1042/BST20110685 PMC3425387

[b9] Gianni D., DerMardirossian C. & Bokoch G. M. Direct interaction between Tks proteins and the N-terminal proline-rich region (PRR) of NoxA1 mediates Nox1-dependent ROS generation. Eur. J. Cell Biol. 90, 164–171 (2011). 20609497 10.1016/j.ejcb.2010.05.007 PMC3013238

[b10] Bögel G. *et al.* Frank-ter Haar syndrome protein Tks4 regulates epidermal growth factor-dependent cell migration. J. Biol. Chem. 287, 31321–31329 (2012). 22829589 10.1074/jbc.M111.324897 PMC3438961

[b11] Fekete A. *et al.* EGF regulates tyrosine phosphorylation and membrane-translocation of the scaffold protein Tks5. J. Mol. Signal. 8, 8 (2013). 23924390 10.1186/1750-2187-8-8 PMC3765130

[b12] Klimczak A. & Kozlowska U. Mesenchymal Stromal Cells and Tissue-Specific Progenitor Cells: Their Role in Tissue Homeostasis. Stem Cells Int. 2016, 1–11 (2016). 10.1155/2016/4285215 PMC4707334 26823669

[b13] Ma S. *et al.* Immunobiology of mesenchymal stem cells. Cell Death Differ. 21, 216–225 (2014). 24185619 10.1038/cdd.2013.158 PMC3890955

[b14] Lu C., Li X.-Y., Hu Y., Rowe R. G. & Weiss S. J. MT1-MMP controls human mesenchymal stem cell trafficking and differentiation. Blood 115, 221–229 (2010). 19901267 10.1182/blood-2009-06-228494 PMC2808151

[b15] Atashi F., Modarressi A. & Pepper M. S. The Role of Reactive Oxygen Species in Mesenchymal Stem Cell Adipogenic and Osteogenic Differentiation: A Review. Stem Cells Dev. 24, 1150–1163 (2015). 25603196 10.1089/scd.2014.0484 PMC4424969

[b16] Mao M. *et al.* The podosomal-adaptor protein SH3PXD2B is essential for normal postnatal development. Mamm. Genome Off. J. Int. Mamm. Genome Soc. 20, 462–475 (2009). 10.1007/s00335-009-9210-9 PMC2759419 19669234

[b17] Hegyi B. *et al.* Regulation of mouse microglia activation and effector functions by bone marrow-derived mesenchymal stem cells. Stem Cells Dev. 23, 2600–2612 (2014). 24870815 10.1089/scd.2014.0088

[b18] Szebeni G. J. *et al.* Identification of Galectin-1 as a Critical Factor in Function of Mouse Mesenchymal Stromal Cell-Mediated Tumor Promotion. PLoS One 7, e41372 (2012). 22844466 10.1371/journal.pone.0041372 PMC3402504

[b19] Eliopoulos N. Allogeneic marrow stromal cells are immune rejected by MHC class I- and class II-mismatched recipient mice. Blood 106, 4057–4065 (2005). 16118325 10.1182/blood-2005-03-1004

[b20] Shi Y. *et al.* Neural cell adhesion molecule modulates mesenchymal stromal cell migration via activation of MAPK/ERK signaling. Exp. Cell Res. 318, 2257–2267 (2012). 22683856 10.1016/j.yexcr.2012.05.029

[b21] Zhang C. Transcriptional regulation of bone formation by the osteoblast-specific transcription factor Osx. J. Orthop. Surg. 5, 37 (2010). 10.1186/1749-799X-5-37 PMC2898801 20550694

[b22] Xu J., Li Z., Hou Y. & Fang W. Potential mechanisms underlying the Runx2 induced osteogenesis of bone marrow mesenchymal stem cells. Am. J. Transl. Res. 7, 2527–2535 (2015). 26885254 PMC4731654

[b23] Liu Z. *et al.* Mediator MED23 cooperates with RUNX2 to drive osteoblast differentiation and bone development. Nat. Commun. 7, 11149 (2016). 27033977 10.1038/ncomms11149 PMC4821994

[b24] Ortuno M. J. *et al.* p38 Regulates Expression of Osteoblast-specific Genes by Phosphorylation of Osterix. J. Biol. Chem. 285, 31985–31994 (2010). 20682789 10.1074/jbc.M110.123612 PMC2952199

[b25] Hishida T., Eguchi T., Osada S., Nishizuka M. & Imagawa M. A novel gene, fad49, plays a crucial role in the immediate early stage of adipocyte differentiation via involvement in mitotic clonal expansion. FEBS J . 275, 5576–5588 (2008). 10.1111/j.1742-4658.2008.06682.x 18959745

[b26] Lehrke M. & Lazar M. A. The many faces of PPARgamma. Cell 123, 993–999 (2005). 16360030 10.1016/j.cell.2005.11.026

[b27] Martella E. *et al.* Secreted adiponectin as a marker to evaluate *in vitro* the adipogenic differentiation of human mesenchymal stromal cells. Cytotherapy 16, 1476–1485 (2014). 24950679 10.1016/j.jcyt.2014.05.005

[b28] Li P. *et al.* Clk/STY (cdc2-like kinase 1) and Akt regulate alternative splicing and adipogenesis in 3T3-L1 pre-adipocytes. PloS One 8, e53268 (2013). 23308182 10.1371/journal.pone.0053268 PMC3537621

[b29] Gruber R. *et al.* Enamel matrix derivative inhibits adipocyte differentiation of 3T3-L1 cells via activation of TGF-βRI kinase activity. PloS One 8, e71046 (2013). 23951076 10.1371/journal.pone.0071046 PMC3741362

[b30] Schild T. *et al.* Expression profiling of microglia and macrophages using novel lipidomic TaqMan(R). Array cards and TaqMan array plates. BioTechniques 46, 315–317 (2009).

[b31] Deutsch M. J., Schriever S. C., Roscher A. A. & Ensenauer R. Digital image analysis approach for lipid droplet size quantitation of Oil Red O-stained cultured cells. Anal. Biochem. 445, 87–89 (2014). 24120410 10.1016/j.ab.2013.10.001

