# Supplementary material for: The scaffold protein Tks4 is required for the differentiation of mesenchymal stromal cells (MSCs) into adipogenic and osteogenic lineages
Source: Sci Rep. 2016 Oct 6;6:34280. doi: 10.1038/srep34280 (PMC5053279; doi:10.1038/srep34280)
Supplement: Supplementary Table S1 [file srep34280-s1.pdf]

The scaffold protein Tks4 is required for the differentiation of mesenchymal stromal cells (MSCs) into adipogenic and osteogenic lineages

M. Dülk, Gy. Kudlik, A. Fekete, D. Ernszt, K. Kvell, J. E. Pongrácz, B. L. Merő, B. Szeder, L. Radnai, M. Geiszt, D. E. Csécsy, T. Kovács, F. Uher, Á. Lányi, V. Vas and L. Buday

Supplementary table S1.

Comparison of the phenotype of our Tks4$^{-/-}$ mouse to other Tks4$^{-/-}$ mice established in other strains and with different strategies

	**Tks4 -/- mice (C57 Bl6 strain)**	**"nee" mice(B10.A-H2h4/(4R)SgDvEg strain)[16]**	**Sh3pxd2b null mice (mixed background)[3]**
**mutation in SH3PXD2b gene:**	deleted 5th and 6th exons resulted in frameshift mutation	frameshift mutation in exon 13.	insertion between 3rd and 4th exons
**smaller body size**	observed	observed	observed
**retardation in movements:**	observed	not tested	not tested
**prominent forehead:**	observed	observed	observed
**prominent deformed eyes:**	observed	observed	observed
**congenital glaucoma:**	not tested	cloudy cornea	observed
**shorter nasal bone:**	observed	observed	observed
**cardiac deficiencies :**	not tested	not tested	observed
**bowing, shortened long bones:**	observed	shortened bones	not bowing
**kyphosis**	observed	observed	observed
**reduced bone mineral density:**	not tested	observed	not tested
**less adipose tissue:**	observed	observed	observed
**fertility:**	infertile	infertile	not tested
**hearing deficiency:**	not tested	observed	observed
