# Supplementary material for: The scaffold protein Tks4 is required for the differentiation of mesenchymal stromal cells (MSCs) into adipogenic and osteogenic lineages
Source: Sci Rep. 2016 Oct 6;6:34280. doi: 10.1038/srep34280 (PMC5053279; doi:10.1038/srep34280)
Supplement: Supplementary Table S2 [file srep34280-s2.pdf]

The scaffold protein Tks4 is required for the differentiation of mesenchymal stromal cells (MSCs) into adipogenic and osteogenic lineages

M. Dülk, Gy. Kudlik, A. Fekete, D. Ernszt, K. Kvell, J. E. Pongrácz, B. L. Merő, B. Szeder, L. Radnai, M. Geiszt, D. E. Csécsy, T. Kovács, F. Uher, Á. Lányi, V. Vas and L. Buday

Supplementary table S2.

List of examined genes of the adipo-differentiated MSCs including gene symbols, short description of the gene functions and assay IDs used by TaqMan Array Plates30.

**Gene symbols:**		**Descriptions:**		**Assay ID**
**Transcription factors:**				
Srebf1		Sterol regulatory element binding transcription factor 1		Mm01138344_m1
Lxra	Nr1h3	Liver X Receptor-Alpha,	Nuclear Receptor Subfamily 1, Group H, Member 3	Mm00443451_m1
Ppard	NR1C2	Peroxisome Proliferator Activator Receptor delta	Nuclear Receptor Subfamily 1 Group C Member 2	Mm01305433_m1
Pparg	NR1C3	Peroxisome Proliferator Activated receptor gamma	Nuclear Receptor Subfamily 1 Group C Member 3	Mm01184322_m1
**Sterol metabolism:**				
Abca1	CERB	ATP-binding cassette transporter A1	ATP-binding cassette sub-family A (ABC1) member 1, Cholesterol Efflux Regulatory Protein	Mm01350760_m1
Lpl		Lipoprotein Lipase		Mm00434770_m1
Cd36		Cd36 scavenger receptor	CD36 antigen, Collagen Type I Receptor, Thrombospondin Receptor	Mm00432403_m1
Hmgcs1		HMG-CoA-Synthase 1	3-hydroxy-3-methylglutaryl-Coenzyme A synthase 1	Mm00524111_m1
Insig1		Insulin induced gene 1		Mm00463389_m1
Stard4		START domain containing 4	StAR-related lipid transfer (START) domain containing 4	Mm00505395_m1
Acat1	MAT	Acetyl-Coenzyme A acetyltransferase 1	Mitochondrial Acetoacetyl-CoA Thiolase	Mm00507463_m1
**Fatty acid metabolism and lipid droplet formation:**				
GK		Glycerol Kinase		Mm00433896_m1
Acadvl		Acyl-Coenzyme A dehydrogenase, very long chain		Mm00444296_m1
FABP4	ALBP	Fatty acid binding protein 4	Adipocyte Lipid-Binding Protein	Mm00445878_m1
FABP5	EFABP	Fatty acid binding protein 5	Epidermal-Type Fatty Acid-Binding Protein	Mm00783731_s1
FADS1	LLCDL1	Fatty acid desaturase 1	Linoleoyl-CoA Desaturase (Delta-6-Desaturase)-Like 1	Mm00507605_m1
FADS2	LLCDL2	Fatty acid desaturase 2	Linoleoyl-CoA Desaturase (Delta-6-Desaturase)-Like 2	Mm00517221_m1
FADS3	LLCDL3	Fatty acid desaturase 3	Linoleoyl-CoA Desaturase (Delta-9-Desaturase)-Like 3	Mm00517643_m1
Scd1	FADS5	Stearoyl-CoA desaturase	stearoyl-Coenzyme A desaturase 1, Fatty Acid Desaturase	Mm00772290_m1
Hadhb	MTPB	Hydroxyacyl-Coenzyme A dehydrogenase beta subunit		Mm00523880_g1
Ucp2	BMIQ4	Uncoupling protein 2	Body Mass Index Quantitative Trait 4	Mm00627599_m1
Adfp	Plin2	Adipose Differentiation-Related Protein, Adipophilin	perilipin 2	Mm00475794_m1
Pla2g4a	CPLA2	Phospholipase A2, group IVA	Calcium-Dependent Phospholipid-Binding Protein	Mm00447040_m1
Ltc4s		Leukotriene C4 synthase		Mm00521864_m1
Alox12		Arachidonate 12-lipoxygenase	Platelet-Type 12-Lipoxygenase	Mm00545833_m1
Alox5ap	FLAP	Arachidonate 5-lipoxygenase-activating protein	Five-Lipoxygenase Activating Protein	Mm00802100_m1
