# Supplementary figures and images for: The scaffold protein Tks4 is required for the differentiation of mesenchymal stromal cells (MSCs) into adipogenic and osteogenic lineages
Source: Sci Rep. 2016 Oct 6;6:34280. doi: 10.1038/srep34280 (PMC5053279; doi:10.1038/srep34280)

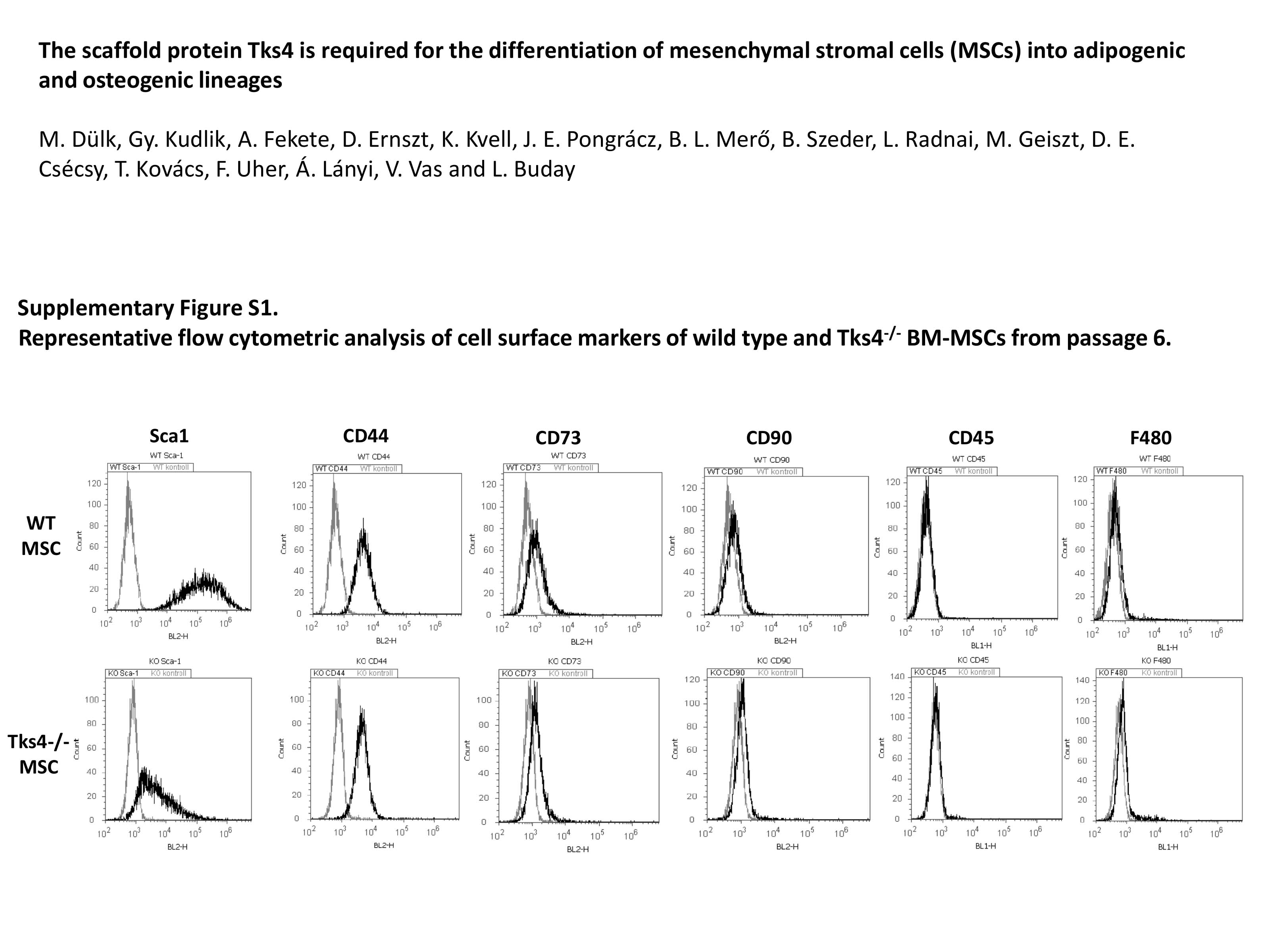

Supplement: Supplementary Figure S1 [file srep34280-s3.jpg]
